# Intrahepatic Arterioportal Fistula With Subsequent Portal Hypertension After Percutaneous Liver Biopsy

**DOI:** 10.14309/crj.0000000000001287

**Published:** 2024-02-28

**Authors:** Makeda Dawkins, Nicholas Cheung, Grigory Rozenblit, David C. Wolf

**Affiliations:** 1 Department of Internal Medicine, Westchester Medical Center, Valhalla, NY; 2 Department of Interventional Radiology, Westchester Medical Center, Valhalla, NY; 3 Department of Gastroenterology, Transplant Hepatology, Westchester Medical Center, Valhalla, NY

## CASE REPORT

**Figure 1.**
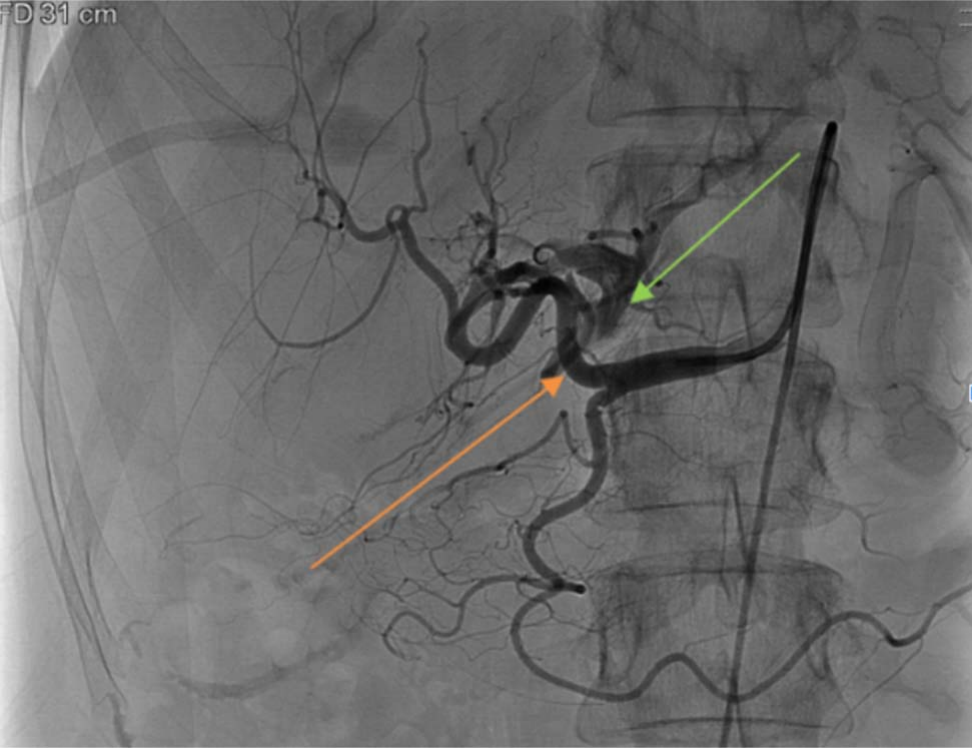
Visualization of the hepatic artery (orange arrow) via angiography with opacification of the left portal vein (green arrow) confirming an intrahepatic arterioportal fistula.

**Figure 2.**
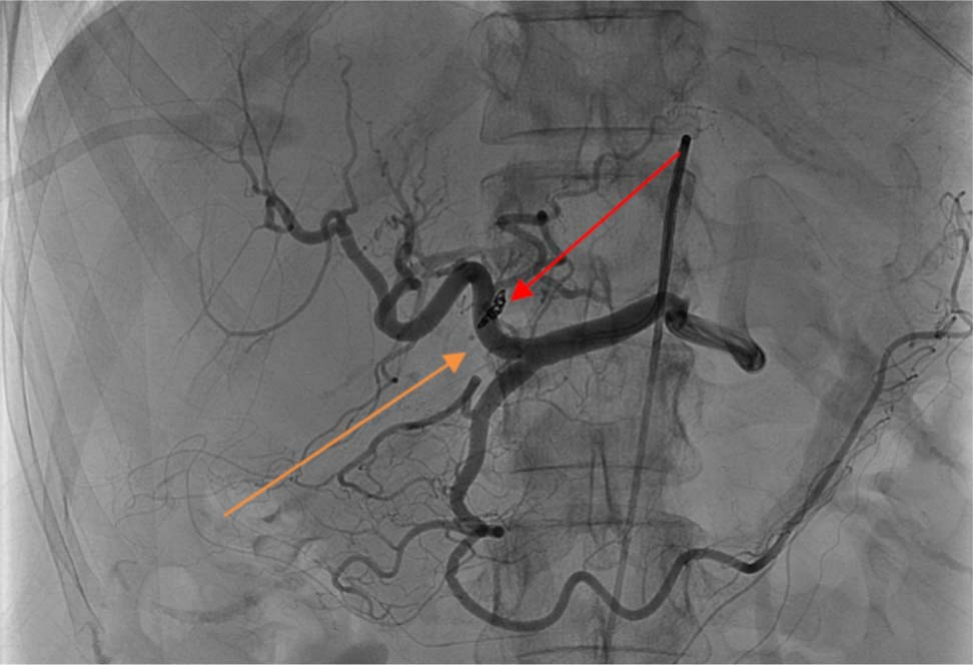
Post-operative angiography indicating successful fistula coil occlusion (red arrow).

A 69-year-old woman presented with transaminitis (aspartate aminotransferase/alanine transaminase 1,072/94 U/L), total hyperbilirubinemia (3.1 mg/dL), elevated international normalized ratio (1.44), and elevated alkaline phosphatase (272 U/L) of unknown etiology. Percutaneous liver biopsy was performed, revealing noncirrhotic autoimmune hepatitis. Immunosuppression was initiated, and liver chemistries improved. Five years later, she developed new onset massive ascites and a recurrent hepatic hydrothorax. Abdominopelvic computed tomography revealed a left lobe intrahepatic arterioportal fistula (APF) with regional parenchymal atrophy. Angiography visualized the hepatic artery (orange arrow) with immediate left portal vein opacification (green arrow) and compromised blood flow to the left lobe, confirming an APF (Figure 1). Catheterization of the proper hepatic and left hepatic arteries was performed with successful APF coil embolization. Postoperative angiography showed successful fistula coil occlusion (red arrow) without compromised perfusion to the right or left lobes (Figure 2). Follow-up evaluation noted resolution of her portal hypertension, ascites, and hydrothorax. Intrahepatic APFs are rare complications of percutaneous and transjugular liver biopsies.^1^ Although smaller fistulas may thrombose spontaneously, larger fistulas and those close to the porta hepatis can cause clinically significant portal hypertension.^2^ Transarterial coil embolization is first-line treatment, with alternatives including *n*-butyl-2-cyanoacrylate embolization, hepatic artery ligation, or partial hepatectomy.^1^

## DISCLOSURES

Author contributions: M. Dawkins is the article guarantor.

Financial disclosure: None to report.

Informed consent was obtained for this case report.

Each author participated in the clinical investigation and/or manuscript generation to a significant extent.

## References

[1] Iwaki T Miyatani H Yoshida Y Matsuura K Suminaga Y. Gastric variceal bleeding caused by an intrahepatic arterioportal fistula that formed after liver biopsy: A case report and review of the literature. Clin J Gastroenterol. 2012;5(2):101–7. 22593771 10.1007/s12328-011-0277-y PMC3328670

[2] Baer JW. Hepatic arterioportal fistula related to a liver biopsy. Gastrointest Radiol. 1977;2(1):297–9. 615827 10.1007/BF02256508

